# Inflammation at the Crossroads: Familial Mediterranean Fever and Cardiovascular Risk

**DOI:** 10.31138/mjr.121025.fcr

**Published:** 2026-04-21

**Authors:** Sejla Karup, Sura Nur Baspinar, Batuhan Ayci, Feyza Nur Azman Tunc, Alperen Akyel, Yelin Guler, Serdal Ugurlu

**Affiliations:** 1 Istanbul University-Cerrahpasa, Cerrahpasa Faculty of Medicine, Istanbul, Türkiye;; 2 Department of Internal Medicine, Istanbul University-Cerrahpasa, Cerrahpasa Faculty of Medicine, Istanbul, Türkiye;; 3 Division of Rheumatology, Department of Internal Medicine, Istanbul University-Cerrahpasa, Cerrahpasa Faculty of Medicine, Istanbul, Türkiye

**Keywords:** Familial Mediterranean Fever, cardiovascular disease, colchicine, inflammation

## Abstract

**Background:**

The understanding between Familial Mediterranean Fever (FMF) and cardiovascular diseases (CVDs) remains unclear. The study’s objective was to investigate the association between FMF and CVD.

**Methods:**

Based on a survey targeting individuals with FMF, the patients diagnosed with coronary artery disease, cerebrovascular disease, and hypertension were considered patients with CVD.

**Results:**

Out of 522 patients, 201 had CVD. The mean ± standard deviation (SD) age was 53.00 ± 6.43 in patients without CVD and 57.60 ± 8.33 in patients with CVD. Hypertension was present in 188 patients (36%), coronary artery disease was observed in 51 patients (9.8%), and 10 patients (1.9%) had cerebrovascular disease. In patients with CVD, diabetes (30.3%) was the most common comorbidity. All patients consumed colchicine. Thirty-five patients with (17.2%) and 25 without CVD (7.8%) had colchicine resistance. 90% continued treatment with colchicine. 25 (12.4%) patients with CVD were using biological agents, as were 18 (5.7%) patients without CVD (p=0.006). The attack period average C-reactive protein (CRP) level was 61.3 (SD=53.1), while in remission, it had an average value of 3.39 (SD=4.28). CRP median was 3.00 (3.65) during attacks in the group with CVD, and 2.00 (2.5) in the group without CVD (p=0.011). CRP median was 2.8 (3.55) in patients with hypertension, and 2.00 (2.7) in patients without hypertension.

**Conclusion:**

FMF does not appear to increase the risk of CVD. Colchicine resistance was associated with the incidence of cardiovascular diseases. Colchicine and anti-interleukin-1 show promise in reducing the risk of cardiovascular diseases in patients with FMF.

## INTRODUCTION

Familial Mediterranean Fever (FMF) is a hereditary autoinflammatory disease that clinically manifests fever, serositis, arthritis, and erysipelas-like erythema. FMF is characterised by mutations in the *MEFV* gene, leading to dysfunctional pyrin protein, which regulates inflammation.^1^ C-reactive protein (CRP), erythrocyte sedimentation rate, white blood cell count, elevated serum fibrinogen concentration, and serum amyloid A are inflammatory markers increased in FMF.^2^ With its anti-inflammatory effects, colchicine is considered the mainstay treatment for FMF. Monoclonal antibodies that target interleukin-1 (IL-1) are frequently employed as an alternative treatment option in colchicine-resistant patients with FMF, disrupting the inflammatory cascade.^3^

Acute inflammation is a physiological response that aids in removing harmful stimuli and initiating tissue repair. In contrast, chronic inflammation has been linked to the development and progression of various diseases, including cancer, autoimmune disorders, and cardiovascular disease (CVD).^4–5^ Inflammation in CVDs is a complex process maintained by the production of proinflammatory cytokines, which activate immune cells and promote endothelial dysfunction. The resulting inflammatory response leads to the formation of atherosclerotic plaques and the progression of CVD.^6^ In addition to the typical symptoms, FMF may be associated with an increased risk of CVD, as chronic inflammation has a fundamental role in the development of atherosclerosis and other CVDs. Because of its anti-inflammatory properties, colchicine has received substantial interest as a potential treatment option for various cardiovascular disorders. Studies have suggested that colchicine^7–8^ and anti-IL-19 have a beneficial effect on CVDs. However, the relationship between FMF and CVD, as well as the interaction between drugs with anti-inflammatory features in FMF and CVDs, remains unclear. To investigate this relationship, we analysed the cardiovascular comorbidities in patients with FMF.

## METHODS

### Survey and Questions

The study enrolled 522 individuals diagnosed with FMF. The diagnosis of FMF was determined using the Tel-Hashomer criteria.^10^ Individuals who met the established criteria for FMF were included in the study. Patients with coronary artery disease (CAD), cerebrovascular disease, or hypertension were classified as having CVD. Colchicine resistance was defined as one or more attacks per month over 3 months or persistent laboratory inflammation between attacks. Our research was a single-centre study conducted in the rheumatology outpatient clinic at Cerrahpasa Faculty of Medicine. The study complies with the Declaration of Helsinki. The research procedure was approved by the Ethics Committee of Istanbul University-Cerrahpasa. Before the survey, each patient provided written informed consent.

Age and gender were the demographic parameters obtained from the patients. The survey encompassed a comprehensive range of data points, namely the year of diagnosis, mutation outcomes, comorbidities, attack occurrences, features of FMF arthritis, familial FMF history, laboratory findings, and treatments for both FMF and other medical conditions. The patients’ survey responses were documented either during their outpatient clinic visit or through a telephone conversation. Attack frequency and patient global assessment scales of disease severity (10-cm visual analogue scale [VAS]) were recorded. VAS ratings were set in the range of 0–10, where 0 represents the lowest and 10 is the highest disease severity.^11^ In our statistical analysis, we employed data obtained from the Turkish Government Statistical Organisation (TUIK) as a control group to ensure robust comparisons and reliable results (https://www.tuik.gov.tr/).

### Statistical Analysis

Statistical analyses were performed using R software v.4.1.2 in RStudio v.2023.12.1. Descriptive statistics were presented as numbers and percentages for categorical variables and as mean ± standard deviation (SD) for numerical variables. The variables were evaluated using Pearson’s chi-square test, Fisher’s exact test, one-way analysis of variance (ANOVA), or the Kruskal-Wallis test, as appropriate.

## RESULTS

The average age of patients with CVD was 57.60 years (SD=8.33) with a female predominance (71.1%), whereas the average age of those without CVD was 53.00 (SD=6.42) with a higher representation of females (59.9%) (**Table 1**).

**Table 1.** Presentation of demographic data of the patients.

	**Patients with CVD (n=201)**	**Patients without CVD (n=321)**
**Age (mean ± SD)^a^**	57.60±8.33	53.00±6.42
**Female**	143 (71.1%)	192 (59.9%)

a p<0.001 and

b p=0.009.

The results revealed that out of the 522 participants, 201 (38.5%) had CVD. One hundred fifty-six patients (29.8%) were diagnosed with one CVD, 42 patients (8%) were diagnosed with two, and three patients (0.6%) were diagnosed with three CVDs. Hypertension was present in 188 patients (36%), while cerebrovascular disease was observed in 10 patients (1.9%), and 51 patients (9.8%) had CAD (**Table 2**).

**Table 2.** Distribution of cardiovascular disease among individuals diagnosed with FMF.

	**Patients (n=522)**
**Hypertension n (%)**	188 (36%)
**Cerebrovascular disease n (%)**	10 (1.9%)
**Coronary artery disease (CAD) n (%)**	51 (9.8%)
**I CVD n (%)**	156 (29.8%)
**II CVD n (%)**	42 (8.0%)
**III CVD n (%)**	3 (0.6%)

171 (53.2%) patients without CVD reported never smoking cigarettes, while 151 (46.8%) were current or former smokers. Similarly, 117 (58.2%) patients with CVD reported never smoking, and 84 (41.8%) were current or former smokers. The average value of the VAS score (0–10) was 2.59 (SD=3.04) for patients without CVD, and 2.73 (SD=3.08) for those with CVD (**Table 3**).

**Table 3.** Cardiovascular comorbidities and medication use in study participants.

	**Patients (n=201) with CVDs**	**Patients (n=321) without CVDs**
**History of smoking n(%)**	84 (41.8%)	151 (46.8%)
**Diabetes^a^** **n(%)**	61 (30.3%)	33 (10.3%)
**Arrhythmia n(%)**	11 (5.5%)	8 (2.5%)
**Colchicine response^b^** **n(%)**	183 (91.0%)	308 (95.9%)
**Colchicine resistance^c^** **n(%)**	35 (17.2%)	25 (7.8%)
**Biological therapy^d^** **n(%)**	25 (12.4%)	18 (5.7%)
**Total number of medications (mean ± SD)**	4.20±2.28	1.74±1.55
**VAS score (mean ± SD)**	2.73±3.08	2.59±3.04

a p<0.001,

b p=0.021,

c p<0.001, and

d p=0.006

Among the comorbidities, diabetes was the most frequent in patients with CVD, affecting 61 (30.3%) out of 201 patients, while it affected 10.3% of patients without CVD (p<0.001). Arrhythmia was also identified as a comorbidity in the group with CVD, affecting 11 (5.5%) patients (**Table 3**). No significant differences were observed for smoking history (41.8% vs. 46.8%, p = 0.29) or arrhythmia (5.5% vs. 2.5%, p = 0.09).

All patients consumed colchicine for FMF. Among the patients with CVD, 35 (17.2%) exhibited resistance to colchicine, while 25 (7.8%) patients without CVD showed similar resistance (p<0.001). Twenty-five (12.4%) patients with CVD were using biologic agents, as were 18 (5.7%) patients without CVD (p=0.006). Patients with cardiovascular disease (CVD) exhibited significantly higher colchicine resistance (17.2% vs. 7.8%, p = 0.002), and use of biologic therapy (IL-1 inhibitor) (12.4% vs. 5.7%, p = 0.021), compared with those without CVD. The average number of prescribed medications in patients without CVD was 1.74 (SD=1.55), while patients with CVD were prescribed an average of 4.20 (SD=2.28) medications (**Table 3**). Five percent of patients had amyloidosis, and none of the patients had cardiac involvement. During attacks, the average CRP level was 61.3 (SD=53.1), while in remission, it had an average value of 3.39 (SD=4.28) (p<0.001). CRP median was 3.00 (3.65) during attacks in the group with CVD, compared to 2.00 (2.5) in the group without CVD (p=0.011). CRP median was 2.8 (3.55) in the group with hypertension, compared to 2.00 (2.7) in individuals without hypertension (p=0.042). The average colchicine dosage was 1.34 (SD=0.442). 90% of individuals continued treatment with colchicine.

In univariate analyses, diabetes (OR = 3.83, 95% CI 2.37–6.19, p < 0.001), colchicine resistance (OR = 2.45, 95% CI 1.39–4.32, p = 0.002), and biologic therapy use (OR = 2.35, 95% CI 1.13–4.87, p = 0.021) were significantly associated with cardiovascular morbidity. In the multivariable logistic regression model, diabetes (aOR = 3.12, 95% CI 1.88–5.19, p < 0.001) and colchicine resistance (aOR = 2.10, 95% CI 1.11–3.97, p = 0.023) remained independent predictors of cardiovascular morbidity. Biologic therapy use was not independently associated (aOR = 1.31, 95% CI 0.61–2.83, p = 0.48). Increasing age was associated with a higher risk, with each 10-year increment corresponding to a 25% increase in the odds of cardiovascular morbidity (aOR = 1.25, 95% CI 1.02–1.54, p = 0.032). Sex, smoking history, arrhythmia, and IL-1 inhibitor therapy did not show independent associations (**Table 4**).

**Table 4.** Multivariable logistic regression for predictors of cardiovascular morbidity in FMF patients.

**Variable**	**^a^OR**	**95 % CI**	**p value**
**Age (per 10-year increase)**	**1.25**	1.02–1.54	**0.032**
**Male sex**	1.12	0.66–1.91	0.66
**Smoking history**	0.86	0.56–1.31	0.47
**Diabetes**	**3.12**	1.88–5.19	**<0.001**
**Colchicine resistance**	**2.10**	1.11–3.97	**0.023**
**Arrhythmia**	1.91	0.78–4.65	0.15
**Biological therapy (IL-1 inhibitor)**	1.31	0.61–2.83	0.48

a OR: adjusted odds ratio.

In **Table 5**, a comparative analysis was conducted between the TEKHARF study and our study, which included a sample of 522 participants. Regarding hypertension, the TEKHARF Study had a total of 6401 cases, with 2955 individuals (46.1%) affected. In our study, hypertension was present in 188 participants (36%) (p<0.001). For coronary artery disease, the TEKHARF Study reported 7457 cases, with 587 individuals (7.8%) affected. In our study, 51 patients had CAD (9.7%). In terms of cerebrovascular disease, the TEKHARF Study had 7457 cases, with 302 individuals (4%) affected. In our study, cerebrovascular disease was present in 10 participants (1.9%) (p=0.016) (**Table 5**).

**Table 5.** Comparative analysis of findings between the TEKHARF study and our study.

	**TEKHARF**	**Our study (n=522)**
**Hypertension^a^**	6401/2955 (46.1%)	522/188 (36%)
**Coronary Artery Disease**	7457/587 (7.8%)	522/51 (9.7%)
**Cerebrovascular Disease^b^**	7457/302 (4%)	522/10 (1.9%)

a p<0.01,

b p=0.016

After age adjustment, the standardised prevalence ratio (SPR) for hypertension was 1.38 (95% CI, 1.12–1.70, p = 0.003), for CAD 1.48 (95% CI, 1.03–2.13, p = 0.034), and for CVD 0.68 (95% CI, 0.35–1.31, p = 0.25). Following adjustment for sex, smoking status, diabetes, and arrhythmia, none of these associations remained statistically significant. In the fully adjusted multivariable model, the adjusted odds ratios (aORs) were 1.09 (95% CI, 0.82–1.45, p = 0.49) for hypertension, 1.22 (95% CI, 0.84–1.78, p = 0.28) for CAD, and 0.79 (95% CI, 0.42–1.47, p = 0.44) for CVD (**Table 6**).

**Table 6.** Adjusted comparison of cardiovascular comorbidities between FMF and TEKHARF cohorts.

**Comorbidity**	**Adjustment type**	**Adjusted estimate**	**95% CI**	**p value**
**Hypertension**	Crude prevalence	36.0% vs 46.1%	—	**<0.001**
	Age-adjusted (SPR)	**1.38**	1.12–1.70	**0.003**
	Sex-adjusted (SPR)	1.21	0.94–1.56	0.13
	Smoking-adjusted (SPR)	1.16	0.88–1.51	0.27
	Diabetes-adjusted (aOR)	1.15	0.86–1.54	0.35
	Arrhythmia-adjusted (aOR)	1.10	0.82–1.47	0.51
	Fully adjusted (age, sex, smoking, diabetes, arrhythmia)	1.09	0.82–1.45	0.49
**Coronary artery disease**	Crude prevalence	9.7% vs 7.8%	—	0.120
	Age-adjusted (SPR)	**1.48**	1.03–2.13	**0.034**
	Sex-adjusted (SPR)	1.36	0.96–1.92	0.078
	Smoking-adjusted (SPR)	1.29	0.90–1.83	0.16
	Diabetes-adjusted (aOR)	1.26	0.88–1.82	0.20
	Arrhythmia-adjusted (aOR)	1.22	0.85–1.76	0.29
	Fully adjusted (age, sex, smoking, diabetes, arrhythmia)	1.22	0.84–1.78	0.28
**Cerebrovascular disease**	Crude prevalence	1.9% vs 4.0%	—	**0.016**
	Age-adjusted (SPR)	0.68	0.35–1.31	0.25
	Sex-adjusted (SPR)	0.72	0.38–1.35	0.31
	Smoking-adjusted (SPR)	0.75	0.40–1.40	0.36
	Diabetes-adjusted (aOR)	0.78	0.41–1.48	0.45
	Arrhythmia-adjusted (aOR)	0.76	0.40–1.43	0.40
	Fully adjusted (age, sex, smoking, diabetes, arrhythmia)	0.79	0.42–1.47	0.44

SPR: standardised prevalence ratio; aOR: adjusted odds ratio.

## DISCUSSION

The intricate relationship between FMF, CVD, and colchicine resistance reveals a pressing need for enhanced treatment strategies. This study confirms a significant prevalence of CVD among FMF patients, with hypertension and CAD being the most frequently observed conditions (**Table 1, Table 2**). Notably, colchicine resistance was markedly higher in patients with CVD (17.2%) compared to those without CVD (7.8%), suggesting that insufficient colchicine response may be a critical factor in increasing cardiovascular risk. Elevated CRP levels during FMF attacks (mean: 61.3 mg/L) and in a group with hypertension further underscore the role of inflammation in exacerbating hypertension and other cardiovascular complications.^12–13^

Colchicine remains the cornerstone of FMF management, primarily through its inhibition of the NLRP3 inflammasome and reduction of IL-1β production. Colchicine’s effectiveness in cardiovascular disease management was demonstrated in studies showing its benefits across different patient populations. Among patients with type 2 diabetes and a recent myocardial infarction, a daily dose of 0.5 mg of colchicine significantly reduced cardiovascular events.^14^ In our study, diabetes and colchicine resistance emerged as independent predictors of cardiovascular morbidity among patients with FMF, even after adjusting for demographic and clinical factors. The strong association between diabetes and cardiovascular comorbidity aligns with the well-established role of metabolic dysfunction in atherogenesis and systemic inflammation. The independent association of colchicine resistance with cardiovascular morbidity may reflect a subgroup of patients with more severe or persistent inflammatory activity, despite standard therapy. Similarly, a randomised trial highlighted its impact on cardiovascular disease, revealing that patients with chronic coronary disease who received 0.5 mg of colchicine once daily had a significantly lower risk of cardiovascular events compared to those who received a placebo.^8^ However, its efficacy is limited in colchicine-resistant patients, as reflected by persistent inflammation that heightens susceptibility to CVDs.^15^ This underscores the importance of using adjunctive therapies to control inflammation in colchicine-resistant populations better. Biological agents, particularly IL-1 inhibitors, have demonstrated promising results for colchicine-resistant FMF patients.^16–19^

In our cohort, biological agents were more commonly used by FMF patients with CVD compared to those without CVD, reflecting a targeted approach to managing severe inflammation in high-risk individuals (**Table 3**). Although biologic therapy users initially showed higher rates of cardiovascular comorbidities, this association did not remain significant after adjustment, suggesting that biologic use may be a marker of disease severity rather than a direct contributor to cardiovascular risk. Age was also a significant factor, consistent with the expected age-related increase in cardiovascular risk. Other variables, including sex, smoking, arrhythmia, and IL-1 inhibitor therapy, were not independently associated with cardiovascular morbidity (**Table 4**). The role of biological agents in addressing colchicine resistance and reducing cardiovascular risk is increasingly evident. Persistent flare-ups and elevated CRP levels in colchicine-resistant patients necessitate aggressive anti-inflammatory strategies. By targeting IL-1, biological agents offer precise modulation of the inflammatory cascade, addressing both systemic and cardiovascular implications of FMF.^9,20^ The integration of biological agents into the management of colchicine-resistant FMF patients not only enhances disease control but also holds the potential for mitigating the broader cardiovascular burden in this population. Evidence from the CANTOS trial supports the use of IL-1 inhibitors in reducing inflammation and lowering the incidence of recurrent cardiovascular events, particularly in individuals with elevated high-sensitivity CRP.^9^ Elevated CRP levels during attacks highlight the inflammation-driven vascular endothelial dysfunction and hypertension that contribute to CVD risk.^21^ Serum CRP levels have crucial prognostic significance in patients with cardiovascular diseases as a marker of low-grade inflammation and are also associated with long-term prognosis in patients with coronary artery disease.^22^

This study highlights the association between colchicine resistance and elevated cardiovascular risk in FMF patients. The higher prevalence of CVD, coupled with persistent inflammation as indicated by elevated CRP levels, underscores the critical need for adjunctive therapies such as IL-1 inhibitors. These agents have shown significant potential in controlling inflammation and reducing cardiovascular events, particularly in colchicine-resistant individuals. Future research should aim to optimise treatment protocols, ensuring better outcomes for FMF patients at heightened risk for CVD. When comparing the findings of this study to the general Turkish population as reported in the TEKHARF study, notable differences emerge. Although the prevalence of hypertension and cerebrovascular disease was significantly lower in our FMF cohort, the prevalence of CAD was slightly higher (**Table 5**). These variations likely stem from the persistent inflammatory environment characteristic of FMF, even during colchicine treatment. Demographic factors further contextualise the findings. Among FMF patients with CVD, the average age was 53 years, with a pronounced female predominance (**Table 1**). These data emphasise the importance of personalised treatment strategies tailored to demographic and clinical characteristics, with special attention to female patients, considering CVD the leading cause of female death.^23^ In the comparative analysis between our FMF cohort and the TEKHARF population, the crude prevalence of hypertension and cerebrovascular disease differed significantly between the two groups. However, after adjustment for demographic and clinical confounders, these differences were no longer statistically significant. The age-adjusted standardised prevalence ratios suggested a modest increase in hypertension and coronary artery disease among FMF patients, but these associations disappeared following full multivariable adjustment for age, sex, smoking status, diabetes, and arrhythmia. In the fully adjusted model, none of the cardiovascular outcomes, including hypertension, coronary artery disease, or cerebrovascular disease, remained significantly associated with FMF (**Table 6**). These findings indicate that conventional cardiovascular risk factors, rather than FMF itself, account for the observed variations and that FMF does not appear to independently increase cardiovascular disease risk.

## LIMITATIONS

The study’s retrospective design might have added biases and confounding variables.

## CONCLUSION

FMF itself does not appear to increase the risk of cardiovascular disease directly; however, elevated CRP levels in this patient group play a crucial role in cardiovascular risk. Persistent high CRP levels, particularly during FMF attacks, may contribute to vascular endothelial dysfunction and the development of CVD. This underscores the importance of managing systemic inflammation effectively, as elevated CRP acts as a key mediator linking FMF-related inflammation to cardiovascular complications. Anti-inflammatory treatments, including colchicine and IL-1 inhibitors, are essential for controlling inflammation and potentially reducing CVD incidence in FMF patients.

## Data Availability

Not applicable.
